# Investigation of Cerebral White Matter Changes After Spinal Cord Injury With a Measure of Fiber Density

**DOI:** 10.3389/fneur.2021.598336

**Published:** 2021-02-22

**Authors:** Vincent Huynh, Philipp Staempfli, Robin Luetolf, Roger Luechinger, Armin Curt, Spyros Kollias, Michèle Hubli, Lars Michels

**Affiliations:** ^1^Department of Neuroradiology, University Hospital of Zurich, Zurich, Switzerland; ^2^Spinal Cord Injury Center, Balgrist University Hospital, University of Zurich, Zurich, Switzerland; ^3^MR-Center of the Psychiatric University Hospital and the Department of Child and Adolescent Psychiatry, University of Zurich, Zurich, Switzerland; ^4^Department of Psychiatry, Psychotherapy and Psychosomatics, Psychiatric Hospital, University of Zurich, Zurich, Switzerland; ^5^Institute for Biomedical Engineering, University and ETH Zurich, Zurich, Switzerland

**Keywords:** spinal cord injury, diffusion weighted imaging, diffusion tensor imaging, fiber density, white matter pathways, sensorimotor function

## Abstract

Remote neurodegenerative changes in supraspinal white matter (WM) can manifest after central lesions such as spinal cord injury (SCI). The majority of diffusion tensor imaging (DTI) studies use traditional metrics such as fractional anisotropy (FA) and mean diffusivity (MD) to investigate microstructural changes in cerebral WM after SCI. However, interpretation of FA readouts is often challenged by inherent limitations of the tensor model. Recent developments in novel diffusion markers, such as fiber density (FD), allows more accurate depictions of WM pathways and has shown more reliable quantification of WM alterations compared to FA in recent studies of neurological diseases. This study investigated if FD provides useful characterization of supraspinal WM integrity after SCI in addition to the traditional DTI readouts. FA, MD, and FD maps were derived from diffusion datasets of 20 patients with chronic SCI and compared with 19 healthy controls (HC). Group differences were investigated across whole brain WM using tract-based spatial statistics and averaged diffusion values of the corticospinal tract (CST) and thalamic radiation (TR) were extracted for comparisons between HC and SCI subgroups. We also related diffusion readouts of the CST and TR with clinical scores of sensorimotor function. To investigate which diffusion markers of the CST and TR delineate HC and patients with SCI a receiver operating characteristic (ROC) analysis was performed. Overall, patients with an SCI showed decreased FA of the TR and CST. ROC analysis differentiated HC and SCI based on diffusion markers of large WM tracts including FD of the TR. Furthermore, patients' motor function was positively correlated with greater microstructural integrity of the CST. While FD showed the strongest correlation, motor function was also associated with FA and MD of the CST. In summary, microstructural changes of supraspinal WM in patients with SCI can be detected using FD as a complementary marker to traditional DTI readouts and correlates with their clinical characteristics. Future DTI studies may benefit from utilizing this novel marker to investigate complex large WM tracts in patient cohorts with varying presentations of SCI or neurodegenerative diseases.

## Introduction

Spinal cord injury (SCI) characteristically results in impairment of the central nervous system and presents with varying clinical characteristics, e.g., loss of motor and sensory function due to disruption of efferent and afferent pathways (1, 2). Traumatic SCI inevitably causes damage to white matter (WM) and mechanical destruction of glial cells and axons at the spinal cord level (1). However, beyond the immediate focal cord damage progressive degeneration in both antero- and retrograde directions of major WM tracts [e.g., the corticospinal (CST)] from the injury site and accompanying neuroplastic changes have been also revealed at supraspinal levels (3–5).

Recent neuroimaging studies including SCI patients have shown macrostructural alterations within the primary sensorimotor cortices, thalamus and frontal regions (3, 6, 7). In addition, current diffusion tensor imaging (DTI) studies have reported microstructural changes within the cerebellum, brainstem regions and large WM tracts (8–14). These DTI studies have provided insight on the impact of SCI in brain WM integrity, e.g., neurodegeneration, as indirectly measured and inferred by fractional anisotropy (FA) and mean diffusivity (MD), respectively (15–18). Whilst FA measures the preferential direction of water movement within tissue architecture, i.e., water molecules diffuse along the direction of tightly packed axons, MD measures the degree of water diffusion. In comparison to healthy controls (HC), SCI patients commonly show decreased FA and increased MD of large WM fibers, i.e., corpus callosum, anterior cingulum, superior and inferior longitudinal fasciculus (12, 19), regions of the CST and primary motor cortex (8, 20), as well as brainstem to midbrain regions (9). Furthermore, two cohorts of patients with a sensorimotor complete SCI and a poor recovery after a 6 months follow-up, showed decreased FA in the cerebellum (10) and primary motor cortex (11). In contrast, patients with an incomplete SCI and a favorable motor recovery at 6 months post-SCI showed similar WM integrity compared to healthy controls (HC) (10, 11). Accordingly, studies that investigated correlations between clinical characteristics and WM integrity reported that SCI patients with better sensory and motor function also showed higher FA values of the posterior thalamic radiations (TR) (13) and pathways of the CST (10), respectively.

However, DTI readouts, such as FA and MD, require careful interpretation when inferring the microstructural integrity of WM in patient cohorts (16). An inherent limitation of the tensor model is the inability to characterize complex fiber orientations, i.e., crossing or “kissing” fibers, which may confound the quantitative changes of FA (21). This problem is addressed with recent optimized methods such as spherical deconvolution (21, 22), which significantly improved the resolution of multiple fiber orientations within a voxel and the tractography method overall (22). Nevertheless, the extraction of reliable quantitative measures from diffusion weighted imaging remains challenging. Recent advances in tractography algorithms (23), microstructural diffusion models (24) and techniques for optimizing tractograms (25) have established a new parameter commonly referred as fiber density (FD). This marker is related to the volume of the intra-axonal compartment per unit of tissue volume, and is therefore sensitive to the volume of a fiber bundle (26, 27). According to the work by Daducci et al., and Smith et al., an optimal weight for each streamline is determined according to a biologically motivated forward model and the measured diffusion signal (25, 28). Assigning a weight of zero allows the elimination of false positive or improbable connections. In this work, we use the COMMIT framework of Daducci et al. (25). One of the results of the COMMIT optimization are intracellular compartment maps (IC maps), which are a direct measure of and correspond to the FD used in the present study. This measurement provides a better representation of the underlying WM tracts and may overcome limitations of traditional tensor derived measures [for DTI limitations see reviews (24, 29, 30)].

With regard to clinical application, FD has recently shown promise in evaluating cerebral WM integrity in neurological diseases such as amyotrophic lateral sclerosis (31) and schizophrenia (45). Specifically, longitudinal changes of FD were observed in commissural and association fiber tracts in amyotrophic lateral sclerosis patients (31) and correlations of positive symptoms with FD of the TR were reported in schizophrenia patients (45). In contrary, no significant results were observed with FA in these two studies, suggesting that FD could be more sensitive to microstructural WM changes in some clinical cohorts compared to FA.

Therefore, this study aims to investigate if FD provides useful characterization of supraspinal WM integrity after SCI in addition to traditional DTI readouts (FA and MD). Specifically, we explore differences between SCI patients and HC in the integrity of the entire WM and also of specific large WM tracts (e.g., CST). We further assess whether the clinical characteristics of SCI correlate with microstructural changes of WM, in particular major tracts involved in sensorimotor processing, i.e., CST and TR. This is performed by subgrouping the SCI cohort based on their severity, i.e., sensorimotor complete (AIS A) and motor incomplete (AIS C-D) lesions (32), and examining the relationship between sensorimotor function and the microstructural integrity of the CST and TR.

Based on previous studies, we hypothesize that patients with a more severe SCI will show decreased FA and FD, and increased MD of major cerebral WM tracts compared to less severely affected SCI patients and HC. Secondly, we hypothesize that SCI patients with better sensorimotor function will show greater WM integrity of the CST and TR.

## Methods

### Inclusion and Exclusion Criteria

Patients with a SCI were contacted and recruited via fllier advertisements and phone calls from the Spinal Cord Injury Center at Balgrist University Hospital and the Swiss Spinal Cord Injury Cohort Study. The inclusion criteria were: (i) Thoracic (T1-T12), traumatic SCI (paraplegics) and all levels of impairment as measured by the American Spinal Injury Association (ASIA) impairment scale (AIS A–D) (33); (ii) no contraindications for MRI; and (iii) no history or presence of other neurological conditions (e.g., traumatic brain injury). The inclusion criteria for HC were no history of neurological illness or other medical condition. All subjects provided written informed consent prior to the assessments and all procedures described below were in accordance with the Declaration of Helsinki. The study has been approved by the local ethics board “Kantonale Ethikkommission Zürich, KEK” (EK-04/2006, PB_2016-02051, clinicaltrial.gov number: NCT02138344).

### Sensorimotor Assessment in SCI Patients

Sensorimotor function was assessed using the sensory and motor scores from the International Standards for Neurological Classification of SCI (ISNCSCI) (33). Specifically, the sum of lateralised sensory scores (light touch and pin prick) and lower extremity motor scores (LEMS, left and right) were extracted to investigate correlations with lateralised WM fibers, i.e., right and left TR/CST. Upper extremity motor scores were omitted as all SCI subjects had intact motor function above the level of injury.

### MRI Data Acquisition

All subjects' MRI data were obtained using a 3.0 Tesla Philips Ingenia system (Philips Medical Systems, Best, the Netherlands) using a 32-channel Philips head coil. Diffusion weighted images were acquired with the following parameters: repetition time (TR), 12700.87 ms; echo time (TE), 79.9 ms; flip angle, 90°; number of slices, 52; slice thickness, 2.3 mm; field of view (FOV), 258 × 119.6 × 258 mm^3^; matrix, 112 × 110 and a scan time of 16:06 min. Diffusion acquisition was performed with a b-value of 1,500 s/mm^2^ along 62 directions after acquiring b0 images. 3D T1-weighted (T1w) structural images were acquired with a Turbo Field Echo sequence with the following parameters: TR, 8.083 ms; TE, 3.70 ms; flip angle, 8°; number of slices, 160; slice thickness, 1 mm; FOV, 240 × 240 × 160 mm^3^; matrix, 240 × 240 and isotropic voxel 0.97 × 0.98 × 1 mm^3^ and a scan time of 4:53 min.

### Diffusion Data Processing

#### Quality Control

The brain extraction tool in FSL 6.0 software (Oxford University Center for Functional Magnetic Resonance Imaging of the Brain, Oxford, UK; https://fsl.fmrib.ox.ac.uk/fsl/) (34, 35) was utilized to remove non-brain tissue from the diffusion data. Subsequently, the diffusion data was corrected for eddy current and head motion with the eddy tool (36). Diffusion tensor residuals were calculated for every acquired diffusion direction and the nine slices in the whole diffusion dataset with the highest residuals were identified for visual inspection. Furthermore, the MRtrix3 software package was applied to estimate the voxel-wise noise level using the residuals from a truncated spherical harmonics fit. Plots were generated depicting the 12 slices with the highest noise level, four in sagittal, four in axial, and four in coronal direction. In addition, mean signal intensity plots for every diffusion direction and the non-diffusion weighted image were derived and plotted slice by slice in sagittal, transverse, and coronal planes. Peaks in these signal courses often indicate head motion. Based on a subject-wise visual inspection of these signal courses and fitting residuals, a rating was performed on a Likert-type scale by a trained MR physicist (range 0–2; 0 = no motion, 2 = high motion). High motion data was omitted from analysis and data was also visually inspected for artifacts.

### Diffusion Processing and Parameter Calculation

Pre-processing of the remaining diffusion weighted images was performed using FSL 6.0 software (Oxford University Center for Functional Magnetic Resonance Imaging of the Brain, Oxford, UK; https://fsl.fmrib.ox.ac.uk/fsl/) and MRtrix3 software package (Brain Research Institute, Melbourne, Australia, version 0.3.12) according to the procedure in Stämpfli et al. (31). Computation of FD maps within the same datasets were performed as follows: Fiber orientation distribution reconstruction and fiber tractography using the default iFOD2 probabilistic tracking algorithm was performed in MRtrix3 (37). Due to the dynamic seeding strategy within the whole WM, the distribution of streamlines is already approximating the apparent fiber density thereby reducing intrinsic tractography biases. A total of five million fibers were generated per subject. The resulting streamlines were optimized using the COMMIT framework (25) and parameters described previously by Sommer et al., to derive the FD for every subject (38). The derived intracellular compartment fraction corresponds to the FD. Full details were previously reported (31). FA and MD maps were calculated with the FSL package including non-linear co-registration, normalization, warping, and skeleton projection (35).

### TBSS Analysis

TBSS analysis of skeletonised FA, MD, and FD maps was performed with the randomize function in FSL. MD and FD images were analyzed using the “tbss_non_FA” command, this step is the standard algorithm of the TBSS framework for scalar diffusion maps other than FA maps. Thus, MD and FD images were analyzed with the same non-linear registration-, warping-, and skeleton projection operations used during the processing of the corresponding FA images (39). Non-parametric permutation test with 5,000 random permutations and a threshold-free cluster enhancement (TFCE) option was implemented and all statistical maps were corrected for multiple comparisons at *p* = 0.05. A one-way ANCOVA was performed to compare differences of whole brain WM between the SCI cohort and HC with age and sex as covariates of no interest.

#### Extracting FA, MD, and FD Values of Large WM Tracts

Mean FA, MD, and FD values of the left and right TR and CST were exported into Statistical Package for the Social Sciences (SPSS) 24 for statistical analysis.

The anatomical regions of interest (ROIs) defining these major fiber tracts were obtained from a probabilistic tractography atlas (JHU white-matter tractography atlas) (40). In total, the JHU white-matter tractography atlas describes 20 major fiber bundles. The probability-weighted mean FD values were calculated for each of the fiber tracts by multiplying the probability-weighted maps of the individual tract with co-registered FD maps from the TBSS analysis. In other words, the probability tract maps served as ROIs to extract the FA, MD, and FD values within each of the fiber bundles. As opposed to the TBSS method, the ROI approach estimates the integrity of entire ROIs, rather than the central integrity of the tracts only and has the advantage of a higher regional sensitivity as compared to a whole brain, voxel-based method.

### Statistical Analyses

All statistical analyses were performed using SPSS (version 24) for Windows. Shapiro-Wilk test was used to test normality of subjects' demographics and independent *t*-test and Chi-squared tests were performed to assess differences in age and sex between the cohorts, respectively. Three separate (FD, FA, MD) one-way multivariate analyses of covariance (MANCOVA) were implemented to test differences of WM tracts between the whole SCI group and HC, alongside differences between SCI subgroups (AIS A and C-D) and HC. DTI values (FD, FA, MD) of the left and right TR and CST were set as dependent variables and grouping of the subjects (i.e., HC, whole SCI and SCI subgroups) were set as independent variables. *Post-hoc* pairwise comparisons were implemented only on WM tracts showing significant main effect of group (*p* < 0.05). Sex and age were added as covariates of no interest for all analyses. *Post-hoc* pairwise comparisons were corrected with Bonferroni correction at *p* < 0.05.

Partial correlations were implemented to assess the relationship between patients' lateralised sensory and motor scores with readouts of the TR and CST, respectively, correcting for age and sex. Only *p*-values that survived Bonferroni-Holm correction are reported (*p* < 0.05).

Furthermore, to identify which diffusion measure(s) of the CST and TR have the highest sensitivity and specificity for differentiating SCI patients from HC, we performed a receiver operating characteristic (ROC) curve analysis. Each diffusion marker of each tract (FA, MD, and FD) were selected as a test probability variable. A numeric grouping variable was selected to test the amount of sensitivity and specificity of each probability variable for delineating subjects into SCI and HC. The amount of sensitivity and specificity is provided by the area under the curve (AUC) and the range from 0.5 (no discrimination)−1 (perfect discrimination) (0.7–0.8 = acceptable; 0.8–0.9 = excellent; 0.9–1 = outstanding).

## Results

### Demographics

Fifty-four subjects were recruited: 25 HC and 29 SCI patients. Five HC and nine SCI patients were excluded due to motion artifacts. Therefore, 19 HC and 20 SCI patients were included in the analyses. Subject demographics are summarized in Table 1.

**Table 1:** Overview of subject demographics.

**Characteristics**	**HC**	**SCI**	**Differences between**
	**(*n =* 19)**	**(*n =* 20)**	**groups *p***
Age (years)	52.0 + 13.0	57.1 + 10.2	0.271
Sex (F/M)	4/15	4/16	0.935
Neurological level of injury	-	Th1–Th12	-
Time since injury (years)	-	15.5 + 10.0	-
AIS (A-D)	-	11A; 2C; 7D	-
Total sensory score (0–224)	-	147.8 + 37.6	*-*
Lower extremity motor score (0–50)	-	15.9 + 19.0	*-*

*Summary of subjects' information. Data is presented as mean and standard deviations. AIS, ASIA Impairment Scale; A, sensorimotor complete; C and D, motor incomplete; F, Female; M, Male; Th, Thoracic; TSI, Time Since Injury; p = significance value*.

### TBSS Whole-Brain WM Analysis

No significant differences of whole brain FA, MD, and FD maps were observed between the whole SCI cohort and HC (all *p's* > 0.05 TFCE). In addition, no significant differences of whole brain FA, MD, and FD maps were observed between SCI subgroups (AIS A; AIS C-D) and HC (all *p*'s > 0.05 TFCE).

### Microstructural Alterations of Large WM Tracts After SCI

Comparing SCI and HC, between subject effects were observed in FA of the left TR [*F*_(1, 38)_ = 8.36, *p* = 0.007], right TR [*F*_(1, 38)_ = 6.45, *p* = 0.016], left CST [*F*_(1, 38)_ = 5.84, *p* = 0.021], and right CST [*F*_(1, 38)_ = 5.80, *p* = 0.022]. Patients presented with decreased FA of the left and right TR (*p* = 0.007 and 0.016, respectively), as well as left and right CST (*p* = 0.021 and 0.022, respectively) compared to HC (Table 2).

**Table 2:** Significant microstructural differences between HC and SCI patients.

**WM tract**	**Readout**	*p*-value^*^
**HC vs. SCI**		
L thalamic radiations	↓ FA	0.007
R thalamic radiations	↓ FA	0.016
L corticospinal tract	↓ FA	0.021
R corticospinal tract	↓ FA	0.022

*Altered microstructural integrity of the thalamic radiations and corticospinal tract in SCI patients. AIS A, sensorimotor complete SCI patients; AIS C-D, motor incomplete SCI patients; FA, Fractional Anisotropy; HC, Healthy Controls; L, Left; R, Right; SCI, Spinal Cord Injury; WM, white matter. ↓, Decreased in SCI patients; ↑, Increased in SCI patients compared to HC*.

* *Bonferroni corrected at p < 0.05*.

Furthermore, between SCI subgroups and HC, between subject effects were only observed in FA of the left TR [*F*_(1, 38)_ = 4.73, *p* = 0.015] and right TR [*F*_(1, 38)_ = 3.84, *p* = 0.031]. *Post-hoc* pairwise comparisons showed that sensorimotor complete SCI patients (AIS A) present with decreased FA of the left and right TR (*p* = 0.015 and 0.029, respectively) compared to HC. No differences were observed between motor incomplete and sensorimotor complete SCI patients or HC (*p* > 0.05). Overall, FD and MD of the left and right TR and CST were not different between the groups (*p* > 0.05).

### Microstructural Integrity of WM Tracts Correlates With Clinical Scores

**Figure 1:**
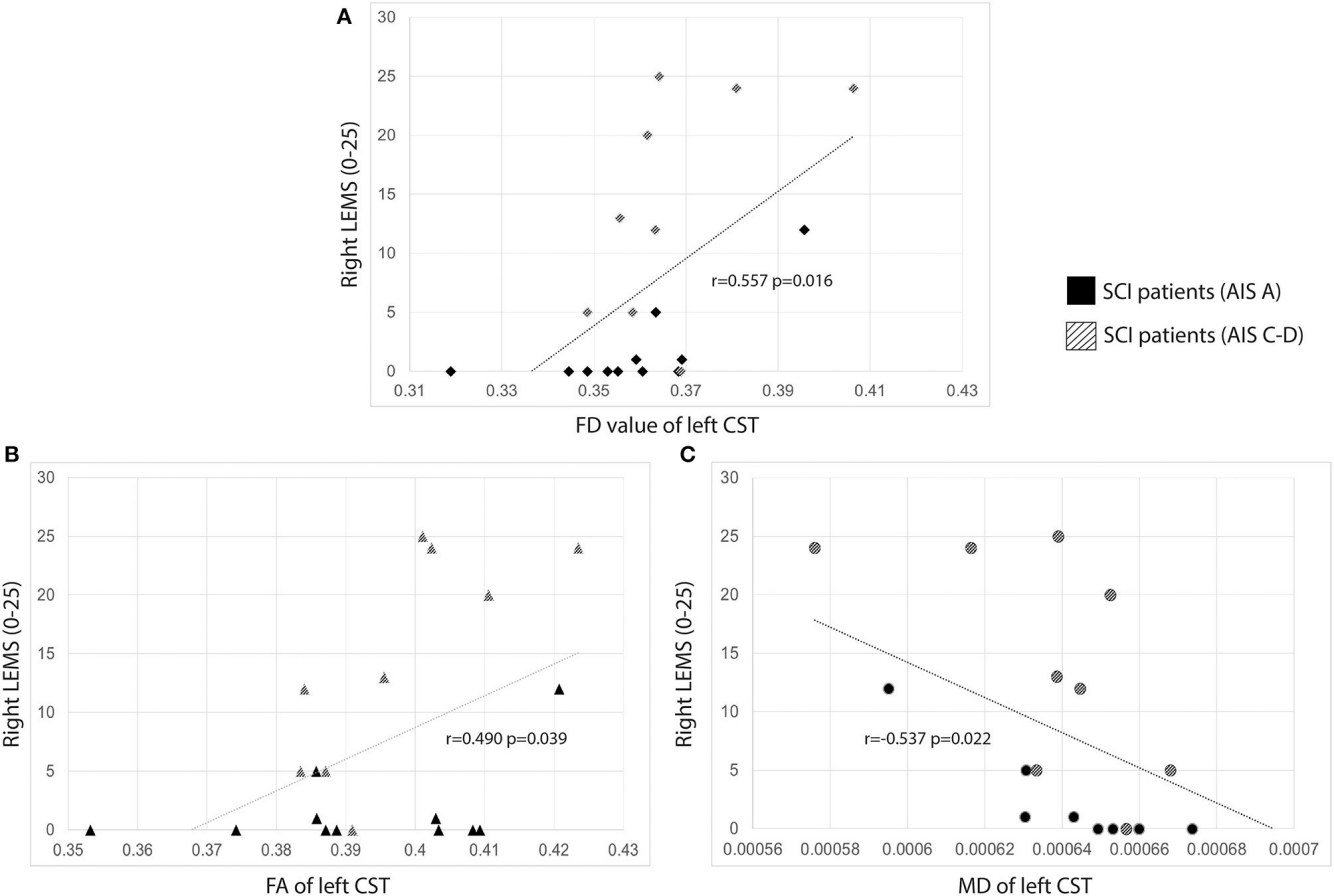
Scatter plots of lateralized LEMS and contralateral CST. Correlations between lateralised LEMS and microstructural readouts of the CST. SCI patients are color-coded based on impairment severity. **(A)** Correlations between lateralised LEMS and FD of contralateral CST; **(B)** correlations between lateralised LEMS and FA of contralateral CST; **(C)** correlations between lateralised LEMS and MD of contralateral CST. Subjects with an AIS A typically had a LEMS of 0. AIS, American Spinal Injury Association Impairment Scale; AIS A, sensorimotor complete; AIS C-D, motor incomplete; CST, corticospinal tract; FA, Fractional Anisotropy; FD, Fiber Density; LEMS, lower extreimity motor score; MD, Mean Diffusivity; SCI, Spinal Cord Injury.

Significant correlations were observed between lateralised LEMS and contralateral TR and CST (Figure 1). Right LEMS correlated positively with FD (*r* = 0.557, *p* = 0.016), FA (*r* = 0.490, *p* = 0.039), and negatively with MD (*r* = −0.537, *p* = 0.022) of the left CST. No other correlations with LEMS were observed. In addition, lateralised sensory scores and microstructural readouts of the contralateral TR did not correlate (*p* > 0.05).

### WM Tracts and Diffusion Measures Showing Highest Sensitivity for Group Classification

The following WM tracts and diffusion measures showed the highest sensitivity for distinguishing subjects into HC and SCI: FA of the bilateral TR, CST, and FD of the bilateral TR (*p* < 0.05) (Supplementary Table 1).

## Discussion

This study explored whether FD, a novel diffusion marker, provides useful characterization of microstructural alterations of cerebral WM in patients with SCI. Although no group differences were observed with FD, FD correlates slightly stronger to the clinical outcome of SCI patients as compared to FA and MD, which could prove valuable in investigating complex WM pathways such as those involved with sensorimotor processing.

### Microstructural Changes of Cerebral WM After SCI

SCI patients showed significant decreases in FA of the TR and CST (Table 2). Also, our ROC curve analysis was able to differentiate between HC and SCI patients based on the FA and FD of the TR (Supplementary Table 1). Previous DTI studies have reported similar decreases in FA of the CST (8, 14, 20), cingulum (13, 19), SLF (12, 19), corpus callosum (13, 19), and TR (13) in various groups of patients with SCI, e.g., cervical vs. thoracic, complete vs. incomplete lesions. Some studies have also reported increases of MD of similar large WM tracts including the CST (8, 9, 13), corpus callosum, cingulum (13, 19), SLF (12, 13, 19), and TR (13). Furthermore, we also observed a significant negative correlation between MD of the CST and TR with patients' motor function. This could signify atrophy, i.e., decreased cell numbers of these remote sensorimotor pathways in patients with more impaired motor function. Whilst decreased FA and increased MD have been associated with impaired WM fiber integrity due to loss of coherent water movement or increased diffusion, careful interpretation is necessary and is dependent on the brain region, cellular composition and the clinical sample studied (16). In general, these DTI markers are sensitive (but non-specific) biomarkers of microstructural architecture and neuropathology (41–43). SCI causes immediate damage leading to inflammatory and degenerative processes, e.g., antero- and retrograde degeneration of WM pathways resulting in neurological dysfunction (1). Previous animal studies investigating microstructural changes at certain locations of the spine after SCI, i.e., epicenter of the injury, proximal and remote to the lesion site, suggest that axonal loss and demyelination occurs in both rostral and caudal directions [see review (44)]. Moreover, previous studies have observed correlations between clinical scores and DTI markers of WM integrity at the brain and spinal level, indicating that these markers are sensitive to the clinical characteristics of SCI patients. In particular, greater sensory scores were correlated with higher FA of the posterior TR (13) and better motor scores were correlated with higher FA within the CST pathway (centrum semiovale) (10) and cervical spinal cord (20).

Overall, our results are consistent with previous studies identifying microstructural abnormalities of cerebral WM fibers in patients with a chronic SCI. In addition, sensorimotor complete SCI patients showed abnormal microstructure of the TR whilst motor incomplete SCI patients showed no observable changes. We also found that patients with a less severe SCI (i.e., greater motor function) showed higher FA and decreased MD of the CST, indicating that a better (preserved) motor function below the level of injury is also associated with greater WM integrity of the major motor pathway (Figure 1).

### FD as a Complementary Marker to Investigate WM Integrity

A substantial benefit of FD is overcoming the limitations of classical DTI readouts, i.e., FA, which is unable to determine multiple fiber orientations within a voxel and therefore a precise anatomical representation of large WM tracts [see reviews (24, 29, 30)]. Better depiction of WM structures is particularly important for complex tracts such as the CST and corpus callosum, where crossing fibers of these two tracts at the centrum semiovale affects the diffusion measures within those voxels (22). FD may therefore be suitable for detecting changes in WM integrity of the CST. This is supported by our identification of a correlation between increased motor function and increased FD of the CST, which were weaker with FA and MD readouts (Figure 1). Furthermore, compared to FA, FD was recently shown to be more sensitive to detect microstructural changes of large WM tracts over a 6 months period in patients with amyotrophic lateral sclerosis (31), alongside WM alterations in patients with chronic schizophrenia (31). In patients with amyotrophic lateral sclerosis, whilst FA remain unchanged, decreases of FD was also observed in the CST, CC, and TR, alongside longitudinal decreases of FD in the CST, TR, corpus callosum, arcuate fasciculus, inferior and superior longitudinal fasciculus and uncinated fasciculus in the left hemisphere (31). In addition, a negative relationship between FD of the TR and higher positive symptoms was observed in patients with schizophrenia, indicating a relationship between clinical characteristics and WM integrity of core structures involved with information processing (45).

SCI patients with better motor function showed greater integrity of the CST with FD showing the strongest correlation compared to FA and MD (Figure 1). Although our ROC analysis was able to differentiate SCI patients and HC based on FD of the TR (Supplementary Table 1), we cannot conclude whether FD provides substantial value in characterizing WM changes as we primarily observed significant group differences of FA in our paraplegic cohort.

### Limitations

In this study, results were based off a relatively small number of patients (*n* = 20) and HC (*n* = 19). This may have decreased statistical power as we did not observe any microstructural changes between the SCI group and HC with whole-brain TBSS analysis. However, no changes in FA or MD have been previously observed in a cohort of sensorimotor complete paraplegics (*n* = 20) (46) and a group of acute patients (*n* = 15) with a heterogeneous SCI (level and severity of injury) (47) compared to HC. It is therefore feasible that widespread WM alterations in our particular cohort is not observable with a whole-brain approach. Nonetheless, with a ROIs approach we observed microstructural changes in sensorimotor WM pathways as observed in prior studies.

### Future Considerations

Future studies are needed to corroborate the validity of FD to identify WM changes following SCI. These studies may benefit by including larger cohorts and subgroups of patients, i.e., paraplegics and tetraplegics with varying severity. Studies that aim to: track neural plastic markers following rehabilitation training and/or therapeutic interventions, or to investigate WM integrity in neurodegenerative diseases, may benefit from utilizing FD to overcome certain limitations of classical DTI readouts. In general, both clinical and animal longitudinal studies are needed to determine whether microstructural abnormalities of cerebral WM coincide with the sequelae of SCI.

## Conclusion

This is the first study to examine a novel diffusion marker (FD) in an SCI cohort. Although our ROC analysis was able to differentiate SCI patients and HC with FD of the TR, significant group differences could only be identified with classical DTI readouts. Importantly, greater microstructural integrity of the CST, as characterized by FD, correlated slightly stronger to the motor function in patients with SCI compared to FA and MD, making FD a potential complementary marker to the classical DTI readouts.

## Data Availability Statement

The datasets used and analyzed during the current study will be made available by the corresponding author upon reasonable request.

## Ethics Statement

The studies involving human participants were reviewed and approved by Kantonale Ethikkommission Zürich, KEK. The patients/participants provided their written informed consent to participate in this study.

## Author Contributions

LM, MH, and VH designed the study and drafted the manuscripts. PS provided major technical contributions to the preprocessing of diffusion datasets. All authors read and revised drafts of manuscripts and approved the final version.

## Conflict of Interest

The authors declare that the research was conducted in the absence of any commercial or financial relationships that could be construed as a potential conflict of interest.
