# Supplementary material for: Investigation of Cerebral White Matter Changes After Spinal Cord Injury With a Measure of Fiber Density
Source: Front Neurol. 2021 Feb 22;12:598336. doi: 10.3389/fneur.2021.598336 (PMC7937730; doi:10.3389/fneur.2021.598336)
Supplement: Supplementary file 1 [file Table_1.DOCX]

# Supplementary Table 1. Significant results of the ROC curve analysis

| *WM tract* | *Readout* | *p-value* | *Area under the curve (AUC)* |
| --- | --- | --- | --- |
| L Thalamic Radiation | FA  FD  MD | 0.005  0.031  0.169 | 0.763  0.703  0.371 |
| R Thalamic Radiation | FA  FD  MD | 0.006  0.031  0.350 | 0.758  0.703  0.109 |
| L Corticospinal Tract | FA  FD  MD | 0.010  0.060  0.109 | 0.742  0.676  0.350 |
| R Corticospinal Tract | FA  FD  MD | 0.020  0.116  0.160 | 0.717  0.647  0.368 |

Table presents the significant diffusion measure(s) and WM tract(s) which show the highest specificity to distinguish between HC and SCI patients. The amount of sensitivity and specificity is provided by the AUC. AUC results range from 0.5 (no discrimination) – 1 (perfect discrimination) (0.7-0.8 = acceptable; 0.8-0.9 = excellent; 0.9-1 = outstanding). *Abbreviations:* AUC – area under the curve; FA – Fractional Anisotropy; FD – Fiber Density; HC – Healthy Controls; MD – Mean Diffusivity; SCI – Spinal Cord Injury.
